# Heat Stroke in the Era of Global Warming: A Call for Urgent Action

**DOI:** 10.5334/aogh.4519

**Published:** 2025-01-20

**Authors:** Ayesha Khan, Manahil Mubeen

**Affiliations:** 1 Dow Medical College, Karachi, Pakistan; 2 Dow University of Health Sciences, Karachi, Pakistan

**Keywords:** Heat stroke, heat‑related illness, climate change, heatwave, preventive measures, public health

## Abstract

Heat stroke (HS) represents a life‑endangering condition that is due to an imbalance between heat generation and dissipation, owing to exposure to hot environments or strenuous exercise. HS is a medical condition that is gaining increased prevalence throughout the world due to a steady rise in temperature, and massive mortalities have been recorded among vulnerable populations. In 2024, extreme heat waves led to increased cases of HS and related fatalities globally, particularly in Karachi, Pakistan. This article reviews the pathophysiology, effects, treatment, and preventive strategies of HS management. Effective management includes prompt on‑site cooling and symptomatic treatment followed by intensive care for severe cases. In keeping heat‑related illnesses low, indoor stay, hydration, and public awareness campaigns play important roles. The urge of the article, therefore, is that HS demands very serious attention from the global arena and its proactive measures should be enforced to avert this medical emergency globally.

Heat stroke (HS), which is considered one of the most fatal diseases, is commonly represented by an unbalanced generation and dissipation of heat due to exposure to a hot environment or strenuous exercise, characterized by a core body temperature > 40°C and multiple organ dysfunction combined with other life‑threatening conditions [1]. HS can be further classified as “classic” or “exertional” depending on the condition’s etiology. Classic heat stroke is observed primarily in sick and immunocompromised individuals, whereas, exertional heat stroke typically affects healthy, young, and otherwise physically fit individuals who collapse during exercise‑heat stress. Both types are generally associated with exposure to hot environments in the absence or presence of strenuous physical activity [2].

As temperatures continue to soar to unprecedented levels, the impacts on human health are becoming increasingly severe and widespread, taking the lives of many in the last many years. The annual heat‑related mortality among individuals over 65 years of age increased by around 68% in the past 20 years and over one‑third of heat‑related deaths in the last 30 years are directly linked to climate change [3]. Due to these hazardous climate changes, In 2018 only, Extreme heat exposure resulted in a loss of 296,000 lives worldwide [4]. Similarly, During the summer of 2022, around 61,672 heat‑related deaths occurred in Europe [5]. In 2023 a substantial increase in heat‑related illnesses (HRI) globally was reported and a total of 119,605 HRI emergency department (ED) visits were recorded only across several US regions [3, 6]. As 2024 is predicted to be among the warmest years in almost two centuries [3], currently, many parts of the world including Europe, India, Saudi Arabia, and North America are experiencing intense heat waves and a warning regarding increased health risks from heat exposure has been issued [7]. South Asian regions are also disproportionately affected by extreme heat, and low‑income populations have inadequate access to cooling facilities, healthcare, and safe water, so they are at high risk of HRI. As a consequence of, the significantly high temperature of about 50 °C, a high number of HRI cases are being reported in Karachi, Pakistan. On 24 June 2024, more than 1500 victims of heatstroke were treated at different hospitals in the city. The days‑long heat wave also killed more than two dozen people, with approximately 20 victims of heatstroke reported on a single day in June at Civil Hospital, out of which eight patients died [8]. More unfortunate is that the city hosts around 200 ventilators only [9], which resulted in complete chaos because many hospitals refused to admit sick and intubated patients because of the unavailability of intensive care units.

Given the alarming statistics on heatstroke, we call for the urgent attention and implementation of proactive measures to prevent it from becoming a “global medical emergency.” This article highlights the pathophysiology, effects, treatment, and preventive strategies for heatstroke. Understanding these aspects is essential for mitigating severe health impacts and reducing the incidence of heat‑related illnesses, particularly in vulnerable populations of developing countries such as Pakistan.

Heat stroke is characterized by severely elevated body temperature with central nervous system dysfunction as the key hallmark symptom that could manifest as epilepsy, delirium, agitation, or coma at the time of collapse [10]. Severe complications, particularly related organ dysfunction, are also common in heat stroke and include gut ischemia, blood clots in the small intestine and stomach, acute kidney injury, cytoplasmic protein clumps in the spleen, and skeletal muscle injury (rhabdomyolysis). Rhabdomyolysis may further induce systemic complications that could include hyperkalemic cardiac arrest, local occurrence of compartment syndrome, and lethal disseminated intravascular coagulopathy. Exacerbations related to untreated heat stroke are renal failure, lactic acidosis, pulmonary edema, psychosis, consumptive coagulopathy, hematuria, and other metabolic diseases [10].

Given the serious complications of HS, it is necessary to effectively manage it. The approach to the management of heat stroke can be divided into primary, secondary, and tertiary prevention.

## Primary Prevention (Reducing Risk Factors for HS)

### Individual

To minimize the number of lives that have been tragically claimed by this heat stroke in Karachi and globally, for that matter, a multifactorial approach focusing on prevention, awareness, and modern treatment options is necessary. As a preventive measure, people should stay indoors, hydrate adequately (at least 2–3 L of water per day), wear light‑colored and breathable clothes, and frequently take baths. Those who work outdoors should dampen the skin using a wet cloth or spray. Individuals at high risk such as the elderly, pregnant patients, and children should be monitored [11].

### Community/Societal

More and more trees should also be planted to mitigate the root cause of heat stroke—“global warming” [11, 12]. Urban planning and infrastructure improvements, such as increasing green spaces, planting trees, and designing buildings with better heat insulation, can lower urban heat island effects and reduce overall heat exposure, reducing the risk of heatstroke.

### Global

Planting trees also contributes to reducing greenhouse gas (GHG) emissions and can contribute to lowering heat levels globally [11, 12]. Acclimatization, or gradually increasing the work hours in a hot environment, is another preventive approach that should be adopted in hot climatic areas globally to allow a person to work harmlessly at the levels of heat that were unbearable for them earlier [12, 13]. Moreover, by detecting areas at high risk of extreme heat occurrences, global monitoring can help with primary prevention of heatstroke by facilitating proactive resource allocation, public health planning, and early warnings to reduce exposure and avoid heat‑related illnesses.

## Secondary Prevention (Early Detection and Intervention)

### Individual

Teaching individuals only about preventive measures is not enough. There is a need to educate every native—including children—about identifying risk factors. Campaigns should emphasize the importance of knowing the early signs of heat stroke, such as excessive sweating, dizziness, and confusion, and adequately spread knowledge regarding symptom recognition in oneself and others. These campaigns should aim to make everyone self‑sufficient in carrying out necessary first‑response actions, such as transporting the affected to a cooler environment, providing instant hydration, and reaching the hospital promptly.

### Community/Societal

Continuous awareness campaigns can help develop a society that is well‑informed to identify signs of HS and act accordingly. Also, community‑based health initiatives should include guidance from local physicians to raise awareness about the potential risk of some medications that can increase susceptibility to heat stroke [12]. Patients and clinicians should carefully evaluate medication regimens in communities living in hot environments. Rather than completely avoiding certain medications such as anticholinergics, diuretics, beta‑blockers, and psychotropic drugs, clinicians should assess the risk‑to‑benefit ratio for each patient. Adjustments in dosages or timing, close monitoring for early symptoms of heat intolerance, and counseling on proper storage during heat waves are essential strategies. Public awareness campaigns can include information on medications that may degrade or lose efficacy in extreme heat, such as inhalers, insulin, and EpiPens, and guidance on their proper storage. Additionally, proactive discussions about symptoms indicating medication‑related heat sensitivity—such as dizziness, excessive sweating, and confusion—can help reduce adverse outcomes [13].

### Global

Standardized guidelines and protocols for the surveillance of vulnerable populations and occupational health standards are needed to mitigate heat‑related risks worldwide.

## Tertiary Prevention (Managing the Impact of HS Through Treatment and Rehabilitation)

### Individual

Patients with heatstroke are treated symptomatically and conservatively. Heatstroke treatment begins on‑site with cardiopulmonary resuscitation (CPR) according to advanced cardiovascular life support (ACLS) protocol, administering oxygen, and monitoring core body temperature with cooling methods such as cold‑water immersion for exertional heatstroke or conductive/evaporative cooling for classic heatstroke. Isotonic saline intravenous (IV) is given for fluid replenishment, while benzodiazepines are administered for seizure control. Immediate evacuation to an emergency department (ED) is essential for classic heatstroke, whereas exertional heatstroke patients should be cooled first [11, 14].

In the emergency room, the core temperature is continuously monitored and lowered by cooling suits and cold fluids with avoidance of antipyretics and dantrolene use. Initial seizure management is done with benzodiazepines or phenytoin, and detailed laboratory testing must be performed, including complete blood count, urinalysis, blood cultures, and renal and hepatic function tests. Circulatory support, where fluid replacement is given, can be provided along with vasopressors if needed [11, 14].

In the intensive care unit, full monitoring and cooling are continued, along with other supportive therapies for heart failure, acute kidney injury, encephalopathy, rhabdomyolysis, coagulation abnormality, acute respiratory distress syndrome, hepatic failure, ECG changes, and systemic inflammatory response syndrome. Treatments may include CPR, extracorporeal membrane oxygenation, invasive hemodynamic monitoring, fluid management, dialysis, continuous veno venous hemofiltration, hyperventilation, and medications specific to each condition for the stabilization of the patient and to prevent multiorgan failure [11, 14].

### Community/Societal

The advanced treatment approaches that have proven effective in the USA for reducing HRI cases should be implemented in communities, such as installing “cooling centers” that provide air‑conditioned environments to vulnerable populations [14, 15].

### Global

Lastly, to manage the impact of HS through treatment globally, we urge the government to look into constructing dedicated medical units specifically equipped to handle emergency epidemics with necessary medical supplies and healthcare staff. During such emergencies, the immense increase in patient load compromises the treatment of already admitted patients. In addition to this, the limited number of tertiary care hospitals further exacerbates the situation. Therefore, international emphasis must be put on capacity building in low‑resource settings.

In conclusion, we urgently call for exploring additional preventive measures and treatment options because, despite currently available treatment options, hundreds of people lose their lives each year.
